# Supplementary material for: Are carbon emissions trading and green financial instruments synergistic? -Comprehensive quantitative research based on content analysis
Source: PLoS One. 2024 Mar 7;19(3):e0298601. doi: 10.1371/journal.pone.0298601 (PMC10919601; doi:10.1371/journal.pone.0298601)
Supplement: S1 Data — (ZIP) [file pone.0298601.s001.zip › Data/地级市Z_F工作报告.zip!/║╙─╧/╫ñ┬φ╡Ω╩╨/└┤╫╘╣1⁄2╓┌║┼í╛┬φ┐╦╩2╛▌═°í┐_2008.docx]

各位代表:

现在我代表市人民政府，向大会作工作报告，请予审议，并请各位政协委员提出意见。

一、2007年政府的工作

(一)项目建设成效显著

，工业速度效益同步提高。年初确定的148个重点项目完成投资92.2亿元，占年度计划的101.2%。其中79个计划竣工项目完成投资60.37亿元，占年度计划的115.8%;16个续建项目完成投资7.79亿元，占年度计划的109.5%;42个计划新开工项目有34个开工建设，在建项目数量和投资额均创历史新高。坚持把工业项目作为重中之重，111个重点工业项目完成投资49.82亿元，占年度计划的111.2%，天方药业盐酸林可霉素、蓝天集团燃气电厂、康茂公司扩建等58个项目已经竣工;顺达化工20万吨醋酸、中集华骏5000辆罐式车、骏化宏福复合肥等29个项目开工建设。积极推进企业战略重组，实现了蓝天与平煤集团、华能集团和昊华骏化与永煤集团的战略合作。工业集聚区建设成效显著，产业吸纳能力不断增强。投入资金1731万元，建设省级、市级工程技术中心7个，重点实验室4个，开发新技术、新工艺210项，开发新产品63个。项目建设和工业工作力度加大，促进了工业经济的发展，限上工业企业比上年增加215户，完成增加值135.1亿元，增长26.6%;主营业务收入486.62亿元，增长47.5%;实现利税56.68亿元、利润36.42亿元，分别增长64%和76.1%。

(二)"三农"工作得到加强

，农村经济全面发展。加大了农业基础建设力度，实施了一批农田水利项目，连续第十次夺得省红旗渠精神杯，新增有效灌溉面积20万亩，新建旱保田24万亩，治理低洼易涝地12万亩，改造中低产田35万亩。在秋季遭受严重洪涝灾害的情况下，粮食总产达到612万吨，其中夏粮总产401万吨，单产、总产均创历史最好水平。认真落实扶持畜牧业发展的政策措施，新建标准化畜牧养殖小区529个，完成畜牧业产值160亿元，占农业总产值的比重达到47.5%。完成成片造林18.1万亩，新建完善农田林网153万亩，绿化通道1138公里，四旁植树2187万株，超额完成了年度目标任务。农业产业化组织达到600个，比上年增加50%;省、市级龙头企业分别比上年增加19家和44家。完成农村劳动力技能培训5.1万人、引导培训13万人，转移劳动力196万人次，实现劳务收入78亿元。

(三)城镇建管力度加大

，人居环境得到改善。完成了中心城区和9个县城总体规划修编工作，扩大了规划覆盖面，提高了规划管理水平。投入资金14.8亿元，完成了中心城区7条市政道路建设和10条景观路的升级改造，新建道路24公里，实施排水工程47.2公里;开工旧城改造项目13个，完成投资8.4亿元，施工面积31.2万平方米;新增绿化面积200.7公顷，绿化覆盖率达到35.4%;中心城区面积发展到48平方公里，人口突破40万人。市区"四城联创"扎实推进，各县城的创建活动深入开展，乡镇环境卫生综合整治成效显著，城镇管理水平得到提高，经营秩序和环境卫生状况发生了明显变化。切实做好户籍改革、撤乡设镇和人口统计工作，城镇人口达到218.7万人，城镇化率25.9%，比上年提高5.3个百分点。

(四)改革开放扎实推进

，发展活力不断增强。国有工业企业改制任务全部完成，企业战略合作取得新突破。行政审批制度改革继续深化，首批6个部门实行职能整合进入行政服务中心办公，审批程序得到简化，服务效率不断提高;组建了公共资源交易中心，规范了各项交易活动。金融改革稳步推进，城市信用社改革基本达到组建商业银行条件，10家农村信用社有9家成功兑付央行专项票据。商贸流通、交通、城建、教育等领域的改革也取得了新成效。坚持把东西合作作为对外开放的主要载体，成功举办了第十届全国东西合作会议，组团参加了第二届中博会和豫港投资贸易洽谈会，全年签约固定资产投资300万元以上合作项目636个，总投资226亿元，已落实项目350个。组织了第三届银企合作洽谈会，签约合作项目41个，合同金额39亿元，落实到位资金15亿元。全市新批境外投资企业16家，实际利用境外资金5764万美元，增长31%，完成出口9300万美元，增长15.2%，均超额完成了省定目标任务。

(五)民生得到进一步改善

，社会事业全面进步。市委、市政府承诺的十五件实事得到较好落实，全年兑付粮食直补、综合补贴等资金5.3亿元;发放"两免一补"资金3.27亿元，受益学生261.1万人;新增农村沼气6万户、通电户8000户、通广播电视自然村310个，解决农村安全饮水21万人。完成"村村通"3137.3公里，所有行政村实现了通水泥(油)路;大广、新驿、驿阳、泌桐4条高速建成通车，实现了县县通高速，4条在建干线公路进展顺利。87个贫困村和7.9万人实现了整村推进脱贫。新增就业64801人，下岗失业人员再就业18874人，其中"4050"人员6793人;城镇登记失业率3.04%，低于省定目标。社会保险征缴面扩大，五大保险参保人数113.27万人;城市低保应保尽保，农村低保扩大到24.6万人，五保户集中供养率达到34.8%。开工建设经济适用房23万平方米、廉租房1万平方米，发放住房补贴1950万元。新型农村合作医疗试点县扩大到5个，参合农民263.8万人，参合率79.6%。发放救灾款物5453.5万元，帮助灾区群众建房7144间。"万村千乡"工程顺利实施，受益群众20万人。提高了农村中小学公用经费标准，建立了校舍维修改造长效机制，新建和改造校舍20万平方米，农村中小学远程教育和课桌凳更新配置工程全部完成;成功申办了职业技术学院，职业教育招生突破4万人。改造乡镇卫生院27所，新建社区卫生服务机构28个，疾病预防控制、急救体系建设和艾滋病防治等工作都取得了新的成效。切实加强人口和计生工作，人口自然增长率5.48‰，继续稳定了低生育水平。广播电视、文化艺术和新闻出版事业不断进步，群众体育和竞技体育健康发展。环境保护和节能减排工作成效显著，9县污水处理厂全部建成投运，5个垃圾处理场一期工程全部竣工;万元生产总值能耗下降4%，化学需氧量和二氧化硫排放量分别下降9%和5%，完成了省定目标任务。统计、人事、审计、旅游、民族宗教、气象、人防、史志、档案、外事侨务等工作，都取得了新的成绩。

(六)社会管理工作得到加强

，社会大局和谐稳定。认真开展"讲正气、树新风"和社会主义荣辱观教育活动，积极推进文明城市、文明单位、文明乡镇、文明社区等创建工作，广大干部群众的精神面貌发生了明显变化。深入开展"双拥"和军民共建活动，军政军民关系得到巩固，再次荣获全国"双拥模范城"称号。坚持依法治市，积极开展普法教育，切实规范行政执法行为，提高了依法行政水平。大力推进政务、村务、厂务公开和城乡居民自治，基层民主建设得到加强。廉政建设和反腐败斗争取得新成效，政风行风继续好转。自觉接受人大法律监督和政协民主监督，认真办理人大代表建议和政协委员提案。继续深化"平安建设"，切实加强社会治安综合治理，严厉打击各种违法犯罪活动，连续四年被评为全省平安建设先进市，人民群众的安全感明显增强;全面落实安全生产责任制，有效防止了重特大事故的发生;妥善解决群众来信来访问题，及时化解各种矛盾，保持了社会大局的和谐稳定。

各位代表，2007年是本届政府施政的第一年，也是我市经济社会全面发展、人民群众得到实惠最多的一年。这些成绩的取得，是省委、省政府和市委正确领导的结果，是市人大、市政协积极支持的结果，也是全市广大干部群众辛勤努力的结果。在此，我代表市人民政府，向各位代表、政协委员和全市人民，向各民主党派、工商联、人民团体和无党派人士，向离退休老同志和社会各界人士，向驻驻部队指战员、武警官兵和公安民警，向所有关心支持驻马店现代化建设的同志们、朋友们，表示崇高的敬意和衷心的感谢!

在肯定成绩的同时，我们也要清醒地看到，当前我市经济社会发展还存在一些困难和问题。主要是:经济总量小，人均水平低，综合竞争力不强;工业骨干企业少，结构层次较低，发展方式粗放，节能减排形势十分严峻;农业基础设施薄弱，农村二、三产业发展滞后，农民持续增收难度较大，新农村建设任务异常艰巨;开放型经济总体水平低，利用外资的规模和层次亟待提升;城镇化水平较低，城乡二元结构矛盾比较突出，县域经济发展滞后且不平衡;社会事业和公共服务投入不足，公共产品短缺，社会保障能力弱，就业压力较大，部分群众生活比较困难;发展环境仍不宽松，少数部门服务意识淡薄、办事效率低下的问题还没有得到根本解决。对于这些问题，我们一定高度重视，进行认真研究，切实加以解决，以更加务实的作风，努力把政府各项工作做得更好，决不辜负全市844万人民的重托和厚望。

二、2008年政府工作的目标任务

今年是全面落实十七大战略部署的第一年，也是我市实施"十一五"规划承上启下的关键一年。今年政府工作的总体要求是:深入贯彻党的十七大和省委八届五次全会、市委二届五次全会精神，深入贯彻落实科学发展观，坚持把发展作为第一要务，继续深化"三项建设"，加快推进"三化"进程，积极推进改革开放，切实转变发展方式，更加重视改善民生，努力促进社会和谐，推动全市经济社会又好又快发展。今年的主要预期目标是:生产总值增长13%，限上工业增加值增长20%以上，城镇固定资产投资增长35%，社会消费品零售总额增长14%以上，财政一般预算收入增长14%，城镇居民人均可支配收入增长10%，农民人均纯收入增长6%，人口自然增长率控制在6‰以内，城镇登记失业率控制在4.5%以内，单位生产总值能耗降低5%，化学需氧量、二氧化硫排放量分别下降4.5%和3.3%。实现上述目标，要努力做好以下七个方面的工作:

(一)继续抓好项目建设

，大力发展工业经济。今年初步安排重点项目152个，年度计划投资100.9亿元。其中计划竣工项目74个，年度投资38.2亿元;续建项目24个，年度投资21.9亿元;计划新开工项目45个，年度投资40.8亿元;前期项目9个。对于这些重点项目，继续实行领导分包责任制，逐项目制定工作方案，明确专人负责，加大督查力度，确保全面完成年度建设任务。突出抓好工业项目建设。今年初步安排重点工业项目120个，年度计划投资69.4亿元。重点抓好吴桂桥煤矿、天方药业那格列奈原料药等67个计划竣工项目，确保按期竣工投产;加快顺达化工36万吨醋酸、中集华骏5000辆罐式车等15个续建项目建设，确保完成年度投资计划;做好国电驻马店热电2×30万千瓦供热机组扩建、平煤蓝天100万吨二甲醚、昊华骏化三聚氰胺尾气综合治理联产60万吨纯碱和氯化铵、白云纸业13.6万吨文化用纸等32个项目的开工准备，确保按期开工建设。继续抓好华润电力2×100万千瓦燃煤机组、昊华骏化合成氨原料路线改造等6个项目的前期工作。同时围绕重点企业、支柱产业和资源优势，积极论证一批大项目、好项目，争取更多项目列入投资计划。大力推进工业结构优化升级。积极改造提升传统产业，努力提高产品的技术含量和附加值。加快能源和煤化工、食品、医药、建材、轻纺基地建设，努力形成规模优势。支持50户重点企业引进战略投资伙伴，加强企业并购和联合重组，支持昊华骏化、平煤蓝天和蓝天集团等企业的上市工作，促使企业做大做强。支持天方药业、蓝天集团、中集华骏等重点企业研发中心和产学研基地建设，引导企业加强科技创新和技术改造，积极培育创新型企业。继续搞好工业聚集区规划布局，努力争取扶持资金，完善园区功能，优化服务环境，积极引导项目和企业进园落户，进一步提高园区的产业聚集能力。重视企业家队伍建设，做到政治上关心、事业上支持、生活上爱护，努力营造企业家成长的良好环境。加强对工业运行的协调指导。高度重视扭亏增盈工作，引导企业加强内部管理，强化市场营销，堵塞跑冒滴漏，提高经济效益。抓好工业运行的监测分析，搞好电力、煤炭、运力、原材料的供求衔接，及时协调解决企业发展中的困难和问题，促进工业经济快速发展。扎实推进节能减排工作。加强9县污水处理厂配套管网建设，确保稳定运营。积极做好市第二污水处理厂的前期工作，力争年内开工建设。加快垃圾处理场建设进度，确保完成年度目标任务。继续淘汰落后生产能力，坚决关停小锅炉、小水泥等"五小"企业。严格执行新建项目节能环保准入标准，防止高耗能、高污染项目上马。突出抓好电力、建材、钢铁、化工、造纸等重点行业的节能工作，支持企业大力发展循环经济。全面落实节能减排统计、监测、考核实施方案，严格实行问责制和"一票否决"制，下决心打好节能减排攻坚战，确保圆满完成目标任务。

(二)切实加强"三农"工作，

扎实推进新农村建设。坚持以农民增收为核心，以产业化经营为突破口，以发展农村经济为首要任务，不断深化农村改革，切实解决民生问题，推动新农村建设取得新进展。加强农业基础设施建设。实施薄山、宋家场、三山等水库除险加固工程，加快小洪河近期治理及老王坡、杨庄滞洪区安全建设，努力完成应急度汛和水毁修复工程。抓好薄山、板桥灌区续建配套和节水技术改造工程建设，继续实施山区小流域治理，加大农业综合开发力度，力争新增有效灌溉面积20万亩、节水灌溉面积3万亩，改善除涝面积10万亩，改造中低产田18万亩，治理水土流失40平方公里。大力发展农村经济。继续落实种粮直补等惠农政策，较大幅度增加农资综合补贴和农机具补贴。继续组织实施好种子工程、沃土工程，突出抓好5个农业综合开发重点县和2个世行项目县建设，力争粮食总产稳定在600万吨以上。加快现代畜牧业发展，全面落实生猪和奶牛生产扶持政策，突出抓好5个重点县、40个重点乡镇和300个养殖小区建设，建立健全疫病防控体系，继续推进养殖业规模化和标准化生产。大力发展特色农业，积极引导县区围绕蔬菜、食用菌、花生、芝麻、花木、生猪、肉牛等优势农畜产品生产，选择1至2个特色产品，加大政策、资金、技术扶持力度，着力培育一批特色明显的专业乡、专业村和专业户，促进区域特色经济快速发展。加大林业生态市、生态县建设力度，完善工作机制，强化工作责任，加大资金投入，完成成片造林35.6万亩、新建绿色通道3014公里、完善农田林网100万亩，确保建成2个以上林业生态县。深入开展"农业产业化推进年"活动，引导各类市场主体参与农业产业化经营，大力扶持农业产业化龙头企业，积极发展各类专业合作组织，力争市级龙头企业发展到200家，年销售收入超亿元企业25家以上，各类农业产业化组织达到800个。继续组织引导好农村富余劳动力转移工作，整合各类培训资金，加大培训力度，打造劳务品牌，力争新增转移就业15万人。分类推进新农村建设。加强组织领导，搞好调查摸底，坚持科学规划、分类指导、分步实施。从环境卫生综合整治入手，利用好上级扶持资金，加快农村饮水、道路、沼气和文化、卫生等基础设施建设，完成通村道路881公里，解决饮水不安全人口20万人，新建农村户用沼气5万座，新增通广播电视自然村310个，新建农村便民超市800个、村标准化卫生室300个，完成"盲点村"电网改造任务，扎实推进900个村的村容村貌整治，切实改善农民生产生活条件。加大扶贫开发力度，完成117个贫困村的整村推进脱贫任务，确保7万农村贫困人口脱贫和解决温饱。

(三)加强城镇建设与管理

努力提高城镇化水平。正确处理新区建设与旧城改造的关系，坚持拉大框架和内涵式发展相结合，进一步完善城镇功能，继续提高城镇的综合承载能力。加强城镇规划工作。完善中心城区道路、供水、商业网点等专项规划，抓好重点地段控制性和修建性详规的编制，力争中心城区详规覆盖率达到38%左右。城区东部以驿城大道建设为契机，规划启动工业集聚区建设，科学布局产业板块，着力打造贯通"两县一区"的工业长廊。城区西部以发展商业、文化教育、住宅为主，逐步完善基础设施和服务功能。按照合理布局、功能完善、方便居民的原则，加强城镇专项规划工作，努力提高规划的科学性和前瞻性。严格城镇规划管理，规范审批程序，加大执法力度，切实维护规划的严肃性和权威性。加快城镇建设步伐。中心城区以"三路三河"建设为重点，抓好驿城大道、天中山大道、铜山大道的延伸改造和练江河、骏马河、冷水河治理工程，完成文化路西段、淮河大道西段等10条道路排水工程建设，加快市区供热、供水、供电等基础设施配套建设，进一步提升城市品位。加快新区建设步伐，完善配套功能，制定优惠政策，鼓励发展高层建筑，提升整体形象。加大旧城改造力度，坚持政府引导、统一规划、市场运作、群众自愿的原则，积极吸引社会资金参与城区改造与建设，治理背街小巷20公里，启动10个城中村改造项目，继续改善人居环境。加快县城和重点镇建设，不断完善公共服务设施，壮大城镇经济，增强城镇吸纳集聚能力。完善通信设施，推进信息技术的应用，努力提高信息化水平。积极创新城镇发展的体制机制，消除发展障碍，做好撤乡设镇、撤镇设办、户籍改革和城镇人口统计等工作，促进城镇化率的不断提高。提高城镇管理水平。深入开展"四城联创"活动，全面落实门前"三包"责任制，建立健全城市管理长效机制，突出抓好城市绿化、美化、亮化和净化，努力实现管理高效化、精细化、规范化，扎实朝着创建目标迈进。引导县城和重点镇以创建工作为载体，继续开展环境卫生综合整治活动，不断提高管理水平，进一步改善城镇面貌。大力发展县域经济。坚持把发展县域经济作为统筹城乡、提升区域竞争力的重要途径，认真落实各项政策措施，加强分类指导，加压驱动，促进企业向城镇和园区集中、生产要素向优势企业集中、人口向城镇集中，努力构建以工业为主导、多种经济形式共同发展的格局，推进县域经济快速发展。

(四)大力发展第三产业

培育新的经济增长点。切实把发展第三产业摆到更加突出的位置，完善政策措施，加强扶持引导，促进第三产业快速发展。大力发展服务业。坚持运用现代经营方式和信息技术，改造提升商贸、餐饮、交通运输等传统服务业，大力发展通信、保险、电子商务、休闲娱乐、市场中介等新兴服务业，拓宽服务领域，提高服务层次，逐步形成以传统产业为基础、新兴产业为支撑的三产发展新格局。继续加大资源整合力度，着力培育一批主业突出、规模较大、竞争力强的大中型物流企业。多渠道筹措资金，加快城镇社区网点建设，健全服务网络，增强服务功能，方便居民生活。加强金融生态环境建设，支持商业银行优化信贷结构，增强金融对地方经济的支持能力。积极吸引有实力的房地产商参与中心城区开发，鼓励优势企业进入县城地产市场，加快房地产业发展。加快城乡市场体系建设。加强城乡商业网点的规划建设，重点抓好"万村千乡"、"双百"、"双进"工程，鼓励商贸、医药、供销等企业在农村发展现代流通业;大力培育专业市场，整合大华、华康、建业等中心城区市场资源，着力培育大型农产品批发市场和流通企业。积极发展文化旅游产业。加快经营性文化事业单位改制步伐，搞好文化资源整合，充分挖掘天中文化内涵，积极培育一批文化龙头企业和知名文化品牌。继续以创建中国优秀旅游城市为载体，重点抓好嵖岈山、铜山湖、薄山湖生态旅游和天中文化旅游、竹沟红色旅游精品景区建设，加强与伏牛山生态旅游圈、华中旅游圈的区域合作，搞好宣传推介，完善服务体系，推动我市旅游产业的快速发展。

(五)深化改革开放，

进一步增强发展的动力和活力。抓住制约发展的关键环节，从解决突出问题入手，不断增强改革开放的积极性、主动性，努力实现改革开放的新突破。继续深化各项改革。扎实推进企业战略重组和资本运作，完善股权结构，建立现代企业制度，努力实现投资主体的多元化。加快中小企业信用担保体系建设，完善促进企业发展的配套政策，促进非公有制企业提升产品档次和发展质量。深化财税体制改革，加强税源建设，强化税收征管，严格预算管理，保障重点支出，努力提高财政资金的使用效益;继续完善县乡财政管理体制，健全村级组织运转经费保障机制，切实增强基层的供给能力。加快城市信用社改革，争取年内组建城市商业银行;继续深化农村信用社改革，完善农村金融服务体系。全面推进林权制度改革，年内完成60%集体林和宜林地确权发证任务。推进干线公路和农村公路管养体制改革，加快交通企业改制步伐。继续深化商贸流通、公用事业、水利、教育、卫生等领域的改革。努力扩大对外开放。按照国家产业政策，围绕资源、产业和区位优势，搞好项目包装和推介，完善招商机制，突出项目招商、企业招商、以商招商，进一步提高招商的质量和成功率。筹备举办好今年的全国农产品加工业博览暨东西合作投资贸易洽谈会，创新办会形式，提高办会水平。积极参加各种投资贸易洽谈和招商活动，多渠道引进好的项目，争取更多的资金。加强跟踪问效，狠抓项目落实，不断提高签约项目的履约率和资金到位率。认真落实招商引资责任制，加强督促检查，严格考核奖惩，推进招商引资工作再上新台阶。进一步优化出口结构，继续巩固芝麻、花生、生猪、香菇等优势农畜产品出口，不断扩大医药、车辆、纺织、化工等工业品出口规模，力争天方药业、华中正大、上蔡荣光3家企业出口额达到2000万美元以上，昊华骏化、康茂、中集华骏等企业达到1000万美元以上。切实抓好利用外资工作，不断提高利用外资水平。积极开展对外劳务合作，扩大外派劳务规模。

(六)切实解决民生问题，

提高城乡居民生活水平。继续实施积极的就业政策，健全创业服务体系，加大就业援助力度，确保城镇新增就业4.2万人，下岗失业人员再就业1.1万人，其中就业困难人员再就业4000人。扩大城镇职工基本养老保险覆盖面，继续完善省级统筹，做实个人账户，从今年起连续三年提高企业退休人员养老金水平。全面实施城乡免费义务教育，免费向农村义务教育阶段学生提供教科书，从秋季起免除城市义务教育阶段学生学杂费，提高贫困家庭寄宿生生活费补助标准。全面开展城镇居民医疗保险试点，扩大城镇职工基本医疗保险覆盖面;全面推行新型农村合作医疗制度，筹资标准由每人每年50元提高到100元;建立和完善城乡医疗救助制度，加强对药品价格和医疗收费监控，解决群众看病贵的问题。继续提高城乡低保和"五保"供养标准，城市低保人均月补差标准由不低于70元提高到不低于110元，农村低保由不低于30元提高到不低于40元，农村"五保"集中供养率达到40%。加大城镇住房保障力度，开工建设经济适用房60万平方米、廉租住房1万平方米。加强整顿和规范市场经济秩序，完善产品质量和食品安全监督网络，大力实施食品药品放心工程。加强价格监测和监督检查，保证市场供应和物价基本稳定。实施职工带薪休假制度。切实维护农民工的权益。

(七)大力发展社会事业，

切实加强社会建设。加快发展交通事业。重点抓好焦桐高速公路泌阳段、化新高速公路、安信一级公路驻马店段等项目建设，积极支持郑武客运铁路专线建设，加快干线公路和农村路网建设进度，完成县乡道路改造615公里;加快市、县客运场站改造和农村客运场站建设，认真实施建制村通公交工程，尽快建成覆盖城乡的客运网络。努力推进科技进步。优化科技资源配置，完善科技服务体系，加强科技交流与合作，做好先进实用科技成果的引进、推广和应用，组织实施一批科技攻关项目，促进科技成果转化，不断提高科技对经济的支撑能力。优先发展教育事业。逐步建立农村义务教育经费保障机制，推进农村中小学布局调整，新建扩建一批城镇和农村中小学，逐步解决大班额问题，推进义务教育均衡发展;抓好高中阶段教育，大力发展职业教育，推进职业教育办学资源整合。筹建驻马店高等幼儿师范专科学校，加快职业技术学院、黄淮学院建设和发展。积极发展民办教育，鼓励引导社会力量投资幼儿教育、高中教育和职业教育。大力发展文化事业。加快市广电中心、体育中心、妇女儿童活动中心、工人文化宫等重点项目建设，开工建设博物馆、群艺馆;启动实施10个农村乡镇综合文化站建设，大力开展文化下乡活动。繁荣新闻出版、文学艺术和哲学社会科学等事业。加强文化市场管理、重点文物和非物质文化遗产保护及开发利用。实施农民体育健身工程，办好第二届老年运动会。积极做好迎奥运服务工作。全面发展医疗卫生事业。加强城市社区和农村三级卫生服务网络建设，分步推进14所县级医院改造。加快河南友谊肿瘤医院、市中心医院病房大楼前期工作，争取年内开工建设。认真抓好重大传染病预防控制，搞好艾滋病防治帮扶工作。稳步推进医疗卫生体制改革，加快建立覆盖城乡居民的公共卫生服务体系、医疗保障体系和药品供应保障体系。认真落实可持续发展战略。坚持计划生育基本国策不动摇，认真落实各项奖励扶持政策，继续稳定低生育水平，提高出生人口素质，加强流动人口计划生育服务管理，综合治理出生人口性别比偏高问题。强化对水、森林、矿产资源的生态保护，加强对洪河、汝河、黑河流域的水污染综合整治，抓好板桥、薄山水库饮用水源地保护，对重点企业实行污染物排放控制，巩固污染物排放防治成果。加强土地管理，落实土地政策，继续开展土地"三项整治"，不断提高土地利用效率。切实加强社会管理。完善和加强城市社区功能，做好第六届村委会换届选举工作。扎实推进精神文明建设，加强社会公德、职业道德和家庭美德教育，深化文明城市、文明单位等创建活动，抓好和谐社区、和谐单位、和谐家庭创建工作。加大殡葬改革力度，认真落实殡葬改革政策。深入开展普法教育，加强法制宣传，全面提高公民法律素质，推进依法治市进程。全面贯彻党的民族宗教政策，充分发挥宗教界人士和信教群众在促进经济社会发展中的积极作用。深入开展国防教育，支持驻驻部队建设，抓好人民防空和民兵预备役工作。积极开展双拥共建活动，巩固和发展军政军民团结。加强法律援助，依法保障公民特别是妇女、未成年人和残疾人的合法权益。完善应急预案体系和管理体制，有效应对各类突发事件，提高危机管理和抗风险能力。深入开展"平安建设"活动，健全社会治安防控体系，严密防范、严厉打击各类违法犯罪活动，始终保持对刑事犯罪的高压态势，不断增强人民群众的安全感。全面落实安全生产责任制，加大专项检查和执法监察力度，及时排查消除各类安全隐患，坚决防止重特大事故的发生。切实做好群众工作，认真落实信访工作"一岗双责制"和责任追究制，妥善解决群众来信来访问题，有效预防和化解各类社会矛盾，促进社会和谐稳定。

三、加强政府自身建设，不断提高施政水平

驻马店作为欠发达市，我们必须坚持把发展作为第一要务，始终唱响发展主旋律，凝聚发展力量，破解发展难题，形成发展合力，尽快缩小与先进市的差距，加快驻马店平安崛起步伐。而要实现这一宏伟目标，必须切实加强政府自身建设，全面提高施政水平，充分调动各方面的积极因素，进一步在全市上下营造想发展、谋发展和抓发展的浓厚氛围。

(一)坚持科学施政，

大力推进政务公开。切实以建设服务型政府为目标，积极深化行政管理体制和运行机制改革，推动政府职能的转变，协调各种利益关系，促进社会公平正义。继续健全科学决策、民主决策机制，不断完善决策程序，提高科学决策水平。对涉及专业性、技术性较强的重大事项，建立健全专家论证、技术咨询和决策评估机制;对涉及群众切身利益的重大决策，建立健全公示、听证制度;对涉及财权、社会资源分配权等重要职权的行使，主动接受社会监督。要围绕服务事项、服务承诺、收费依据、办事时限、责任追究等关键环节，按照《政府信息公开条例》的要求，不断扩大各类行政管理和公共服务事项的公开范围，努力实现各类行政职权的全过程动态公开和全流程接受监督，全面提高政府工作的透明度，保障人民群众的知情权、参与权和监督权。

(二)坚持以人为本，

努力做到勤政为民。政府的一切权力都是人民赋予的，必须对人民负责，为人民谋利益。各级政府要始终把关注民生、改善民生作为一切工作的出发点和落脚点，把政府的着力点更多地放在群众最关心的热点难点问题上，把关注点更多地放在维护弱势群体利益上，把有限的财力更多地投入到与人民群众息息相关的民生问题上。制定工作措施，要充分了解民情，看是否符合群众的意愿;落实方针政策，要充分依靠群众，征得大多数群众的支持。要善于站在群众的立场上考虑问题，凡是关系群众利益的事，有条件的一定要积极落实，没有条件的要创造条件落实。要集中精力办好市委、市政府确定的15件实事，真心实意为群众做好事、解难题，努力实现好、维护好、发展好广大群众的根本利益，以实实在在的政绩取信于民。

(三)坚持改进作风，

全面提高服务水平。大力倡导说实话、办实事、求实效的工作作风，力戒形式主义。以完善目标管理为重点，健全政府绩效考核体系，加大政务督查力度，明确责任，严格奖惩，把各方面的力量引导到科学发展、和谐发展上来。坚持精简会议和文件，严格控制和规范各种检查、评比、达标活动。加强行政服务中心建设，深化行政审批制度改革，进一步减少审批事项，规范行政行为，扎实推进窗口单位的职能整合，努力提高集中审批办结率。全面推行首问负责制、服务承诺制、限时办结制和办结公告制，切实将"一站式"办公、"一条龙"服务的要求落到实处。继续严肃查处破坏发展环境的行为，特别是对那些执法犯法、吃拿卡要的典型案件，要坚决依法依纪惩处，切实提高政府的公信力和执行力。以对人民高度负责的态度，保持良好的精神状态，奋发有为，迎难而上，努力开创各项工作新局面。

(四)坚持依法行政，

切实加强廉政建设。各级政府都要严格按照法定权限和程序行使权力、履行职责。充分发挥行政监察和审计监督的作用，认真落实执法责任制和执法过错追究制，努力做到有权必有责、用权受监督、违法要追究。自觉接受市人大及其常委会的法律监督、工作监督，市政协的民主监督以及新闻舆论监督和群众监督，广泛听取各民主党派、工商联和无党派人士的意见和建议。把反腐倡廉建设放在突出位置，坚决惩治和有效预防腐败。坚持厉行节约，勤俭办事，反对铺张浪费和奢靡之风，努力建设节约型机关。认真落实廉政建设责任制，严格执行廉洁自律的各项规定。加强公共资源交易中心建设，继续抓好重点领域的监督管理和制度落实，切实做到用制度管权、按制度办事、靠制度管人，全力打造勤政为民、廉洁从政、务实高效的公务员队伍。

各位代表，当前驻马店的经济社会发展已经进入一个新的阶段。圆满完成今年的各项目标任务，责任重大，使命光荣。让我们在省委、省政府和市委的正确领导下，坚持以党的十七大精神为指导，深入贯彻落实科学发展观，解放思想、振奋精神，团结奋进、扎实工作，为促进驻马店经济社会又好又快发展、早日实现全面建设小康社会目标而努力奋斗!
